# Yanggan Yizhong decoction prevents liver metastasis from colorectal cancer by targeting myeloid-derived suppressor cells through the regulation of bile acid metabolism in the gut microbiota

**DOI:** 10.3389/fmicb.2025.1639442

**Published:** 2025-09-15

**Authors:** Hongting Xie, Shijie Zhu, Peng Xue, Feiyu Xie, Leyi Zhao, Xuelei Chu

**Affiliations:** ^1^Department of Oncology, Wangjing Hospital of China Academy of Chinese Medicine Sciences, Beijing, China; ^2^Integrated Traditional Chinese and Western Medicine Department, The Cancer Hospital of the University of Chinese Academy of Sciences (Zhejiang Cancer Hospital), Hangzhou, China; ^3^Graduate School of Beijing University of Chinese Medicine, Beijing, China; ^4^Department of Oncology, Guang'anmen Hospital, China Academy of Chinese Medical Sciences, Beijing, China

**Keywords:** colorectal cancer, liver metastasis, Yanggan Yizhong decoction, gut microbiota, bile acid, immunosuppression

## Abstract

**Introduction:**

Liver metastasis (LM) exhibits a high incidence in colorectal cancer (CRC), yet effective preventive therapies are still lacking. Based on the prophylactic principle of harmonizing the liver and spleen, Yanggan Yizhong (YGYZ) decoction has shown clinical effectiveness in preventing LM. This study aims to explore the active components and underlying mechanisms of YGYZ in the prevention and treatment of LM.

**Methods:**

The components of YGYZ were analyzed using Ultra-High Performance Liquid Chromatography coupled with High-Resolution Tandem Mass Spectrometry (UPLC-HR-MS/MS). The LM mouse model was established through intrasplenic injection of ct26-luc cells to evaluate the effect and safety of YGYZ on LM. Fecal microbiota transplantation (FMT) was performed to create microbiota-altered mice, and liver tissue morphology along with HE staining was utilized to dynamically monitor LM progression. Flow cytometry and inflammatory factor assays were conducted to assess the immune microenvironment (IME) of the liver pre-metastatic niche (PMN). Additionally, 16S rRNA sequencing and bile acid (BA) metabolomics were employed to investigate the role of YGYZ in modulating gut microbiota (GM) and BA. Western blot analysis was performed to identify key targets of YGYZ in the GM-BA-immunity pathway.

**Results:**

UPLC-HR-MS/MS analysis identified 95 compounds in YGYZ, Glycyrrhizic acid, Bergapten, and Icariin as the main compounds. YGYZ and its FMT inhibited LM of CRC with safety, inhibited CD11b+Ly6G+ and CD11b+Ly6C+ cells in the pre-metastatic stage, decreased CD11b+Ly6G+ cells in the metastatic stage, reduced immunosuppressive factors such as Arg-1, TGF-β, and IL-10, and improved the CD4+/CD8+ T-cell ratio, regulating liver PMN. YGYZ also improved the GM structure, particularly decreasing the abundance of Clostridium in the LM mice. For the hepatic BAs profile, YGYZ increased the content of primary BAs—Nor cholic acid (NorCA), Taurocholic acid, Taurochenodeoxycholic Acid, and Tauro β-Muricholic Acid, and secondary BAs—ursodeoxycholic acid (UDCA), with similar trends in FMT, while YGYZ decreased NorCA, α-Muricholic acid, Tauro α-Muricholic acid, and UDCA in the fecal BA profile. YGYZ and its FMT dampened the protein expression of IL-6, STAT3, and pSTAT3, but only YGYZ downregulated kruppel-like factor 15 (KLF15).

**Conclusion:**

YGYZ may prevent LM by remodeling the GM and synergistically inhibiting KLF15 to regulate the enterohepatic BA cycle, and suppressing the proliferation and activation of myeloid-derived suppressor cells through the IL-6/STAT3 pathway, thereby improving IME of liver PMN.

## 1 Introduction

The 2022 International Agency for Research on Cancer report documented 1,926,118 new colorectal cancer (CRC) cases globally, resulting in 903,859 CRC-related deaths (Bray et al., 2024). During the natural progression of CRC, liver metastasis (LM) occurs in 40–50% of patients. Notably, among CRC-related deaths, 49% had predominantly hepatic lesions and 83% had liver involvement (Stewart et al., 2018). Currently, standard treatment for LM from CRC relies on surgery, yet only 10–20% of cases achieve curative resection, with the operative site often becoming a recurrent focus of metastasis (Eng et al., 2022). Furthermore, radiotherapy, chemotherapy, targeted therapies, and immunotherapies are constrained by adverse effects and drug resistance, consequently failing to achieve satisfactory prognoses (Aykut and Lidsky, 2023; Kaviyarasan et al., 2024). LM is associated with unsatisfactory treatment responses and dismal survival outcomes, posing a persistent clinical dilemma. Therefore, prophylactic intervention serves as an essential approach to decreasing LM occurrence and improving survival outcomes.

In 2005, Kaplan et al. first proposed the concept of the pre-metastatic niche (PMN) in Nature, defining it as a tumor-induced microenvironment in specific organ tissues, promoting metastatic colonization prior to the actual dissemination of the primary tumor (Kaplan et al., 2005). The liver’s dual portal-caval circulation system mediates the trafficking of CRC cells and bone marrow-derived components, collectively orchestrating PMN development. Myeloid-derived suppressor cells (MDSCs) are the primary immunosuppressive players in this process, crucially influencing the hepatic PMN (Zhou et al., 2022). Moreover, the liver and colon establish bidirectional communication through biliary metabolism and circulatory exchange. Within this enterohepatic axis, gut microbiota (GM) and bile acids (BAs) demonstrate critical functional interplay with the liver PMN status (Conche et al., 2023).

Rooted in clinical practice continuously, Traditional Chinese Medicine (TCM) employs complex natural formulations that simultaneously modulate multiple biological targets to restore systemic homeostasis. Emerging pharmacological studies and clinical reports support the application of TCM formulations against LM. Yanggan Yizhong (YGYZ) decoction, a representative prescription based on the liver-spleen coordination principle, has demonstrated potential in both preventive and therapeutic clinical applications (Zhao et al., 2020). Using a ct26-luc mouse model, our study aims to elucidate the effects of YGYZ on LM from CRC through comprehensive analyses of GM, BAs, and MDSCs.

## 2 Materials and methods

### 2.1 Chemical components of YGYZ by ultra-performance liquid chromatography-high resolution tandem mass spectrometry analysis

YGYZ was provided by Wangjing Hospital of China Academy of Chinese Medical Sciences, with raw materials sourced from Kangmei Pharmaceutical (China). The composition and dosage of each herb in YGYZ are detailed in Table 1. Herbal components were macerated in 10 volumes of distilled water (1 h), then decocted twice. The combined aqueous extracts were lyophilized to yield a 4.0 g/mL crude extract (in distilled water), stored at 4 °C.

**Table 1:** Composition and content of each herb in YGYZ.

Numbers	Herbs	Amount
1	*Rehmanniae Radix Praeparata*	15 g
2	*Angelica Sinensis Radix*	12 g
3	*Paeoniae Radix Alba*	15 g
4	*Chuanxiong Rhizoma*	9 g
5	*Epimedii Folium*	10 g
6	*Crinis carbonisatus*	10 g
7	*Codonopsis Radix*	20 g
8	*Citri Reticulatae Pericarpium*	10 g
9	*Zanthoxyli Pericarpium*	6 g
10	*Coicis Semen*	30 g
11	*Amom Fructus*	6 g
12	*Glycyrrhizae Radix et Rhizoma*	10 g

For sample preparation, 0.2 g of the herbal extract was dissolved in 50% methanol, centrifuged for 5 min, and filtered through a 0.22 μm membrane to obtain the test solution. Chromatographic separation was performed on a Waters ACQUITY UPLC HSS T3 column (2.1 × 100 mm, 1.8 μm) maintained at 35 °C using a Waters Synapt G2-Si Q-TOF mass spectrometer. The mobile phase consisted of 0.1% formic acid in water (phase A) and acetonitrile (phase B) with a flow rate of 0.25 mL/min. The gradient elution program was as follows: 0–15 min, 100–80% A; 15–50 min, 80–0% A; 50–60 min, 0% A; 60–70 min, 0–100% A. Mass spectrometric analysis was conducted using a Q-Exactive system with an injection volume of 10 μL.

### 2.2 Animal experiments

All animal experiments were approved by the Ethics Committee of the Medical Experimentation Center of the China Academy of Chinese Medical Sciences (Approval No. ERCCACMS21-2302-07). The sample size was determined following the ARRIVE 2.0 guidelines, based on effect size estimation from preliminary experiments and calculation of the model’s coefficient of variation, while adhering to the 3R principles of animal ethics. For information on animal numbers, refer to the figure legends. All experiments used BALB/c mice (male, 6–8 weeks, 18–20 g) that were purchased from Beijing Vital River Laboratory Animal Technology (License No. SCXK (Jing) 2021-0006). The animals were housed in specific pathogen-free facilities with controlled temperature (22 ± 2 °C) and humidity (50 ± 10%). After 7-day acclimation with ad libitum feed/water, mice were maintained under 12:12 light: dark cycles.

Animals were randomly divided into six experimental groups using a random number table. LM models were established using a spleen-preserving technique (Xu et al., 2020) wherein 100 μL (2 × 10^6^ cells/mL) of ct26-luc cell suspension (American Type Culture Collection, United States) was slowly injected into the spleen, while sham-operated controls received equal-volume PBS injections. Following a 3-day recovery period with prophylactic penicillin treatment (40,000 IU, i.p.), six groups received different intervention plans respectively: Group Sham (0.9% saline, 0.1 mL/10 g/d, p.o.), Group Model (0.9% saline, 0.1 mL/10 g/d, p.o.), Group Capecitabine (Cap, H20133365, Jiangsu Hengrui Medicine, China), (150 mg/kg/d, p.o.), YGYZ-high dose (YGYZ-H, 39.78 g /kg/d), YGYZ-medium dose (YGYZ-M, 19.89 g/kg/d), and YGYZ-low dose (YGYZ-L, 9.95 g/kg/d). The clinical dosage of YGYZ was 2.19 g/kg/d (total herb weight per dose was 153 g, based on a human average body weight of 70 kg). The mouse equivalent dose was calculated as 19.89 g/kg/d through body surface area conversion (Nair and Jacob, 2016). Experimental performers were blinded to group allocation until data analysis was completed. All treatments were administered orally in 0.1 mL/10 g volumes. Terminal endpoints included comprehensive evaluations followed by tissue collection.

Fecal microbiota transplantation (FMT) was conducted as previously described (Wu et al., 2021; Zong et al., 2023) with modifications. Before this, we conducted a preliminary experiment and found that a YGYZ-high dose of 39.78 g/kg/d demonstrated the most significant therapeutic effects and safety in mice. Therefore, fresh YGYZ-H donor feces were homogenized in anaerobic PBS (1:10 w/v), centrifuged (800 × g, 3 min), and filtered (70 μm) to obtain a microbiota-rich supernatant (25 mg/mL). Group FMT received 0.25 g/kg/day via daily gavage.

For *in vivo* bioluminescence imaging, mice were intraperitoneally injected with D-luciferin potassium salt (150 mg/kg, ADP104300004809391, Promega, United States) and anesthetized using isoflurane (R510-22-16, Shenzhen Ruiwode Lift Technology, China). Animals were positioned in prone orientation within the imaging chamber of a Bruker *In-Vivo* Xtreme imaging system (Bruker, United States). Signal intensity was quantified with the following spectral representation: red indicating high signal intensity and blue representing background-level signals.

### 2.3 Tests of liver and kidney function

Liver and kidney function were assessed in serum samples using standardized colorimetric kits (Jiangsu Zecheng Biotechnology, China) with the following references: alanine aminotransferase (ALT, 20192400200), aspartate aminotransferase (AST, 20192400201), blood urea nitrogen (BUN, 20192400212), and creatinine (CRE, 20192400214).

### 2.4 Hematoxylin and eosin staining

Liver tissues were immediately fixed and paraffin-embedded, then sliced with a 3 μm thickness and air-dried. Stained with hematoxylin for nuclei and with eosin for cytoplasm after returning to the blue. Finally, dehydrated and sealed for examination by a microscope and image collection.

### 2.5 Immunohistochemical assay

Liver tissues were immediately fixed and paraffin-embedded. Serial sections (3 μm) were cut, deparaffinized in xylene, and rehydrated through a graded ethanol series. Antigen retrieval was performed using sodium citrate buffer (pH 6.0) at 95 °C for 15 min. Sections were then incubated overnight at 4 °C with primary anti-Ki-67 antibody (1:200, BS-23103R, Beijing Boaosen Biotechnology, China) in a humidified chamber. Immunoreactivity was visualized using DAB chromogen, followed by hematoxylin counterstaining and sealed. Ki-67-positive cells were quantified under an Olympus BX53 microscope (Olympus, Japan).

### 2.6 Immunofluorescence staining

Paraffin-embedded liver sections (3 μm) were deparaffinized and rehydrated through graded ethanol. Antigen retrieval was performed using citrate buffer (pH = 6.0) at 95 °C for 15 min. Endogenous peroxidase activity was blocked with 3% hydrogen peroxide for 10 min. Sections were incubated with optimally diluted primary antibodies in a humidified chamber at 4 °C overnight. HRP-conjugated secondary antibodies were applied for 50 min at room temperature (RT). Following microwave-mediated epitope recovery, the third antigen was detected using fluorophore-conjugated secondary antibodies (50 min, RT, light-protected). Nuclear counterstaining was performed with DAPI, followed by autofluorescence quenching (Solution B, 5 min) and mounting with anti-fade medium. Multispectral images were acquired using a fluorescence microscope (Nikon, Japan). Antibodies purchased from BioLegend Corporation (United States) were used for target detection: APC anti-mouse CD3 Antibody (1:200, 100235), PE anti-mouse CD4 Antibody (1:200, 100407), FITC anti-mouse CD8 Antibody (1:200, 140403), APC/Cyanine7 anti-mouse/human CD11b Antibody (1:200, 101225), APC anti-mouse Ly-6G Antibody (1:200, 127613), FITC anti-mouse Ly-6C Antibody (1:200, 128005).

### 2.7 Flow cytometry

Liver tissues were enzymatically digested with 0.25% trypsin solution. The filtered cell suspension was centrifuged and resuspended to obtain single-cell preparations. Cells were stained with the following fluorescently conjugated antibodies from BioLegend Corporation (United States): CD11b, Ly6G, Ly6C, CD3, CD4, and CD8. An unstained control was included for compensation. After 30 min incubation at 4 °C protected from light, cells were washed and fixed in 500 μL PBS containing 1% paraformaldehyde. Flow cytometry (Changzhou Bidak Biotechnology, China) was performed, and FlowJo7.6.1 Software (United States) was used to analyze the results.

### 2.8 Enzyme-linked immunosorbent assay

Liver tissue homogenates were centrifuged, and the resulting supernatants were collected and diluted uniformly. Cytokine levels were quantified using commercial ELISA kits (Beijing Bioassay Systems Biotechnology, China): Arg-1 (arginase-1, MD35262), TGF-β (transforming growth factor-β, MD132594-1), IL-10 (interleukin-10, MD106493), IL-6 (interleukin-6, MD106412). Absorbance was measured at 450 nm using a microplate reader. Cytokine concentrations (Arg-1, TGF-β, IL-10, and IL-6) were quantified in ng/mL or ng/L by interpolating absorbance values against a standard curve generated with recombinant proteins.

### 2.9 16S rRNA sequencing

Fresh fecal samples were collected to extract total microbial DNA, with DNA concentration and purity assessed by NanoDrop (United States). Sequencing libraries were prepared with the TruSeq Nano DNA LT Library Prep Kit (Illumina, United States). Libraries were sequenced on Illumina NovaSeq following standard denaturation (NaOH) and dilution protocols. Microbiome analysis was performed using Quantitative Insights Into Microbial Ecology. Metabolic pathway information was determined through the MetaCyc database.^1^

### 2.10 Metabolomic analysis of BAs

Fresh fecal and liver tissue samples were collected for analysis. BAs profiling was performed using an ACQUITY UPLC^®^ BEH C18 column (2.1 × 100 mm, 1.7 μm) (Waters, United States) coupled to an AB Sciex 6500+ QTRAP mass spectrometer. Mass spectrometric detection employed multiple reaction monitoring (MRM) with optimized transitions for the quantification of individual BAs (Supplementary Table S1).

### 2.11 Western blot

Homogenized tissues were lysed in lysis solution, mixed, and then cells were broken by sonication at maximum power (3 × 10s) and centrifuged (12,000 rpm, 15 min) to collect the supernatant. Protein concentrations were determined using the BCA assay, with samples normalized to 5 mg/mL. Proteins were separated by 10% SDS-PAGE and transferred to PVDF membranes. After blocking with 5% non-fat milk for 1 h at RT, membranes were incubated overnight at 4 °C with primary antibodies (Affinity Biosciences, United States): anti-β-actin (1:5000, AF7018), anti-kruppel-like factor 15 (KLF15) (1:1000, DF12203), anti-STAT3 (1:2000, AF6294), and anti-pSTAT3 (Tyr705, 1:1000, AF3293). Following washes, membranes were probed with HRP-conjugated secondary antibodies (1:5000) for 1 h at RT. Protein bands were visualized using enhanced electrochemiluminescence (ECL).

### 2.12 Statistical analysis

Statistical analysis was performed using SPSS 26.0 (IBM, United States), and graphs were generated with GraphPad Prism 9.0 Software (United States). All continuous variables were first assessed for normality. For multiple-group comparisons of normally distributed data, one-way ANOVA was employed, followed by appropriate *post-hoc* tests. Non-normally distributed data were compared among multiple groups using the Kruskal-Wallis test with *post-hoc* analysis. Microbiome and metabolome data were corrected for multiple comparisons (Benjamini-Hochberg FDR). Correlation analysis was conducted using Pearson’s correlation coefficient. A two-tailed *p* < 0.05 was considered statistically significant for all analyses.

## 3 Results

### 3.1 YGYZ inhibited LM from CRC with a safety profile

**Figure 1:**
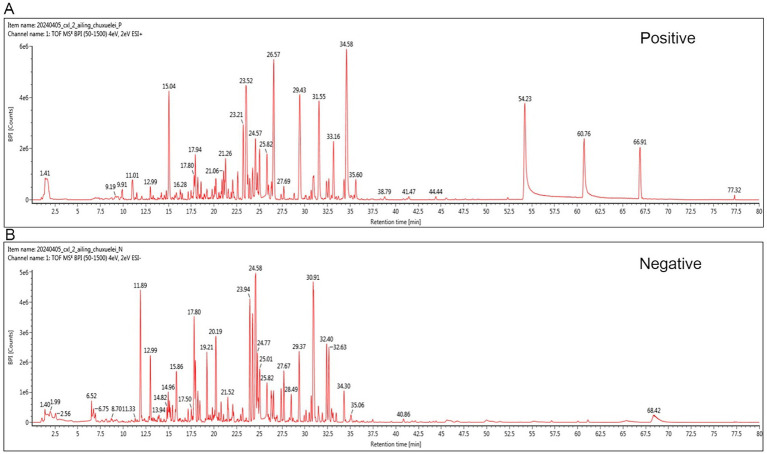
Representative base peak chromatogram of YGYZ in the positive and negative ion modes. **(A)** Positive mode. **(B)** Negative mode.

Phytochemical profiling of YGYZ by UPLC-HR-MS/MS identified 95 compounds (Supplementary Table S2; Figure 1), including 20 flavonoids, 15 phenylpropanoids, 14 organic acids/derivatives, 12 terpenoids, 5 alkaloids, 8 phenolic compounds, 6 oligosaccharide/polysaccharide derivatives, and 15 other classifications. Key representative compounds included Glycyrrhizic acid, Kokusaginine, Icariin, Icaritin, Bergapten, Sagittatoside B, Licurazide, Epimedin C, and Hesperetin.

**Figure 2:**
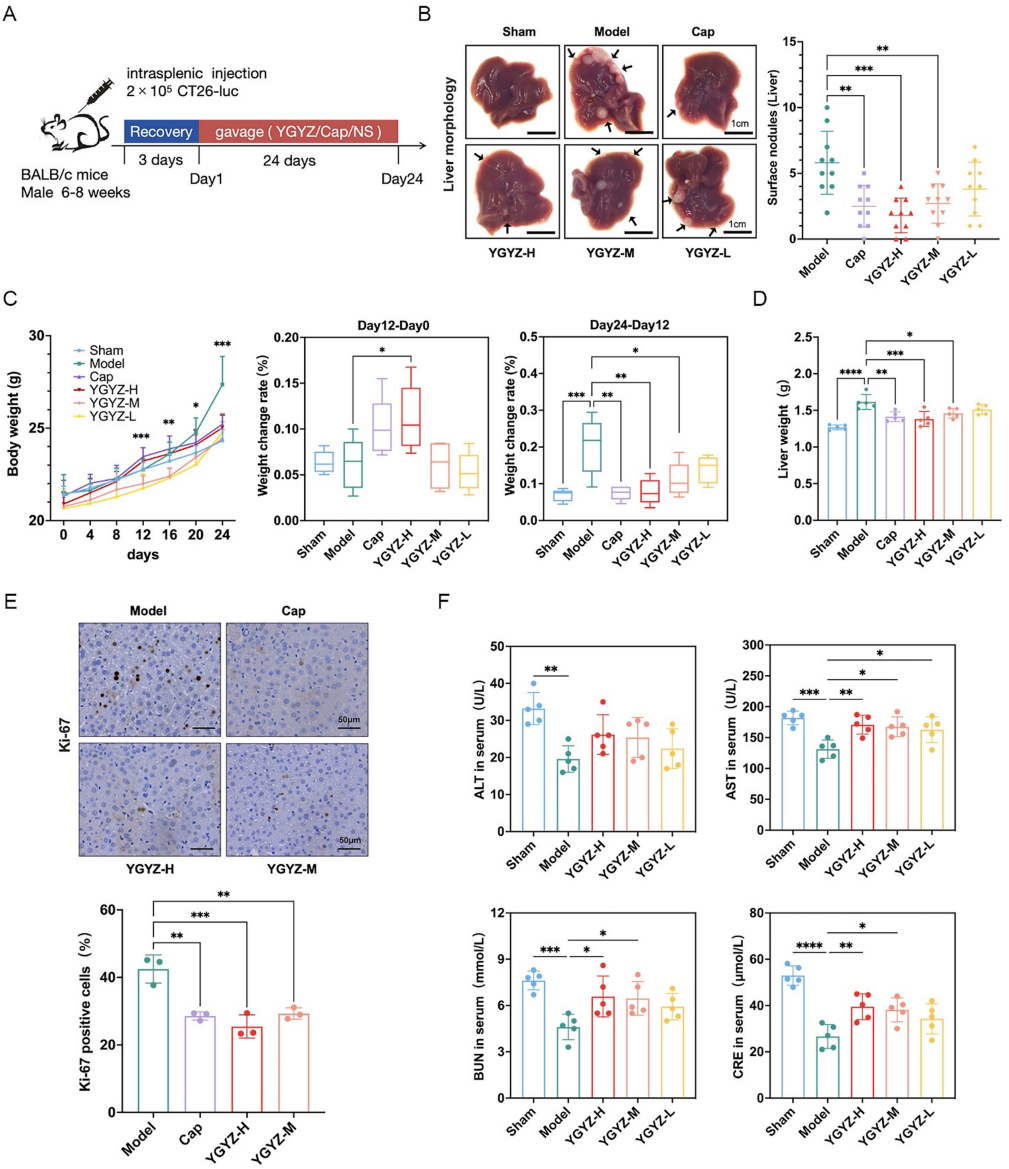
YGYZ inhibited liver metastasis (LM) from colorectal cancer with a safety profile. **(A)** Schematic of modeling and intervention. **(B)** Liver histology and the superficial number of LM (*n* = 10). **(C)** Body weight change from day 0 to 24 and percentage of weight gain from day 0 to 12 and from day 12 to 24 (*n* = 5). **(D)** Liver weight (*n* = 5). **(E)** Ki-67 immunohistochemical staining and percentage of Ki-67 positive cells (*n* = 3). **(F)** Serum ALT, AST, BUN, and CRE levels (*n* = 5). Data are expressed as Mean ± SD. Statistical analysis was performed using one-way ANOVA or the Kruskal-Wallis test, followed by *post hoc* tests. *p < 0.05, **p < 0.01, ***p < 0.001, ****p < 0.0001.

To evaluate YGYZ’s preventive effects on LM, we established the model via intrasplenic injection of ct26-luc cells (Day 0), followed by 24-day YGYZ administration (Figure 2A). Both YGYZ-H and YGYZ-M reduced hepatic metastatic nodules compared to the Model group, with efficacy comparable to Cap (Figure 2B). From day 0 to 12 gavage, the YGYZ-H group exhibited higher body weight gain compared to the Model (Figure 2C). From day 12 to 24 of gavage, Model group gained the faster body weight than Sham, Cap, YGYZ-M, and YGYZ-H groups (Figure 2C). The liver weight of mice in the YGYZ-H, YGYZ-M, and Cap groups was reduced compared with that of the Model (Figure 2D). The proportion of Ki-67-positive cells in YGYZ-H, YGYZ-M, and Cap groups was reduced compared with Model, indicating suppressed tumor proliferation (Figure 2E). All of these results indicated that YGYZ inhibited the progression of LM. Compared with the Model group, YGYZ-H, YGYZ-M, and YGYZ-L increased the AST level, while YGYZ-H and YGYZ-M increased the BUN and CRE levels (Figure 2F). The results suggested that YGYZ preserved liver and kidney functions. Integrated analysis of both anti-metastatic efficacy and safety parameters established YGYZ-H as the most favorable group for follow-up experiments.3.2 YGYZ and FMT inhibited tumor progression and delayed LM.

**Figure 3:**
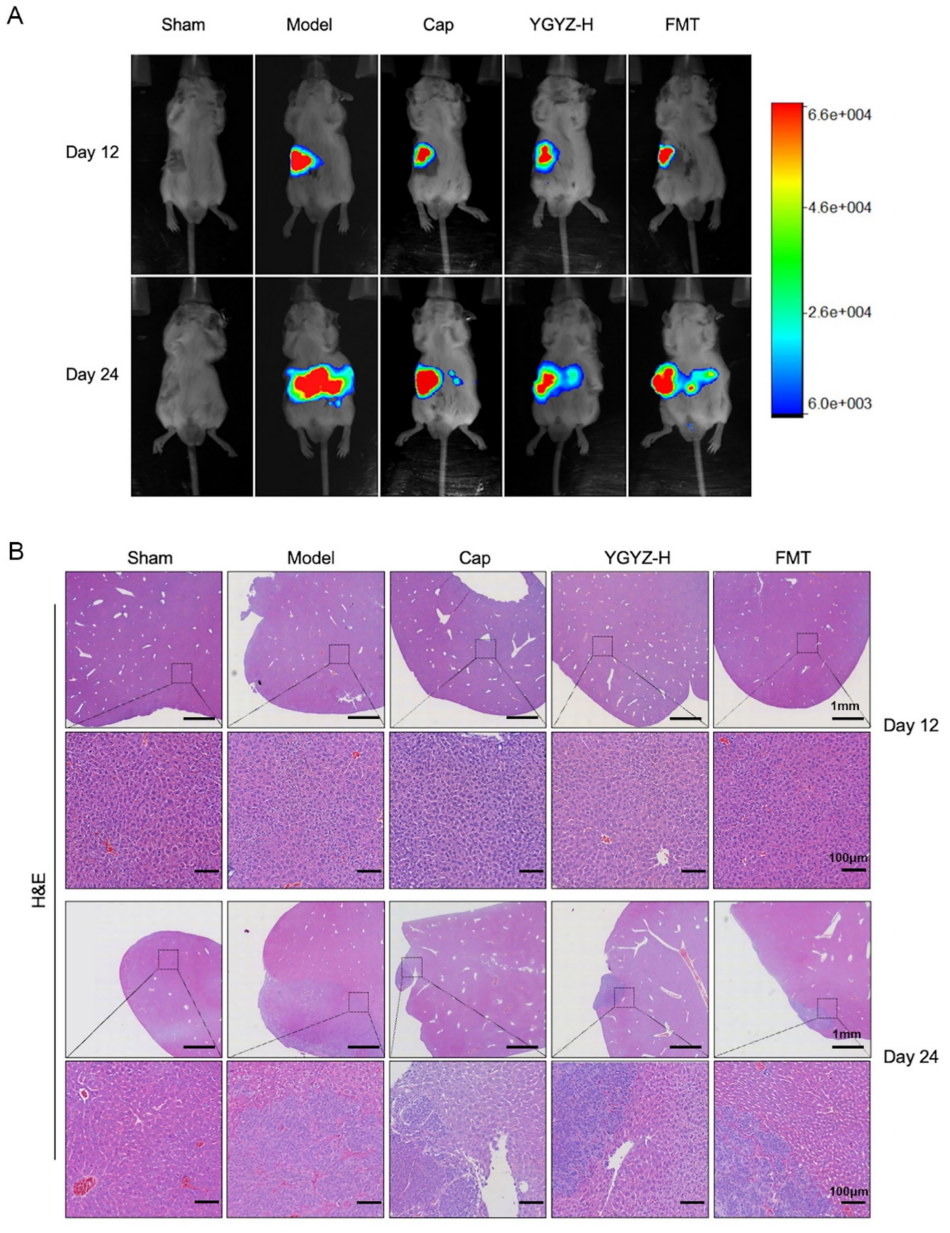
YGYZ and its fecal microbiota transplantation (FMT) inhibited tumor progression and delayed liver metastasis at different disease stages. **(A)** Live imaging of small animals on days 12 and 24. **(B)** HE staining of liver on days 12 and 24.

The progression of LM was dynamically observed by the *in vivo* imaging system, and on the 12th day of intervention, the ct26-luc cells were clustered in the right splenic region, and on the 24th day of gavage, fluorescent signals were observed in the left hepatic region. The strongest fluorescent signals were observed in the Model on both day 12 and 24, and the intensity in the Cap, YGYZ-H, and FMT groups was weaker (Figure 3A), suggesting that all three groups inhibited the proliferation of ct26-luc cells and delayed tumor progression and metastasis.

HE staining revealed distinct metastatic progression phases. At day 12, the liver exhibited no observable metastases across all groups, demonstrating preserved hepatic architecture with orderly hepatocyte cords and intact portal triads. By day 24, the Model developed extensive metastatic lesions characterized by hypercellular tumor colonies displaying malignant features including nuclear pleomorphism, loss of cellular polarity, and prominent tumor vasculature. Therapeutic interventions significantly reduced the metastatic burden versus the Model (Figure 3B). Combining the results of small animal *in vivo* imaging and HE staining, the liver pre-metastatic stage on day 12 and the liver metastatic stage on day 24 could be set to further analyze the immune microenvironment (IME) at each stage.

### 3.2 YGYZ and FMT remodeled the PMN by suppressing MDSCs

**Figure 4:**
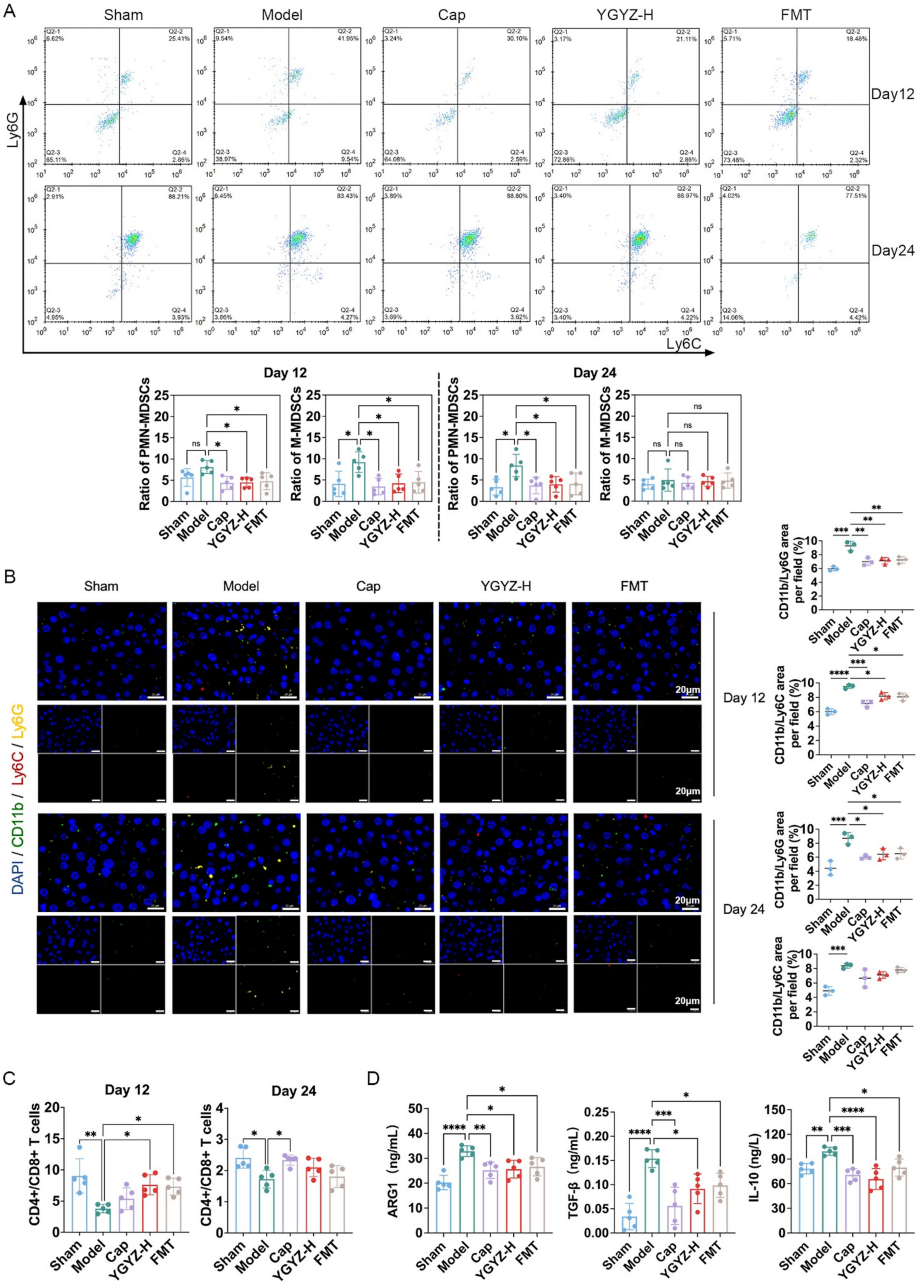
YGYZ and fecal microbiota transplantation (FMT) remodeled the pre-metastatic niche at different stages of liver metastasis. **(A)** Subpopulations of liver MDSCs on days 12 and 24 by flow cytometry (*n* = 5). **(B)** Subpopulations of liver MDSCs on days 12 and 24 by immunofluorescence staining (*n* = 5). **(C)** The hepatic CD4+/CD8+ T cell ratio by flow cytometry (*n* = 5). **(D)** Hepatic ARG1, TGF-β, and IL-10 expression on day 12 by ELISA (*n* = 5). Data are expressed as Mean ± SD. Statistical analysis was performed using one-way ANOVA or the Kruskal-Wallis test, followed by *post hoc* tests.*p < 0.05, **p < 0.01, ***p < 0.001, ****p < 0.0001. ns: no significant difference.

Flow cytometry revealed that during the pre-metastasis period on day 12, the proportions of hepatic PMN-MDSCs and G-MDSCs were reduced in the Cap, YGYZ-H, and FMT groups, compared with the Model. On day 24 (LM period), the proportions of hepatic PMN-MDSCs were reduced in the Cap, YGYZ-H, and FMT groups, with no significant difference in the proportion of G-MDSCs (Figure 4A). Immunofluorescence staining showed that on day 12, the proportions of CD11b+Ly6G+ and CD11b+Ly6C+ cells were increased in the Model, which were decreased in the Cap, YGYZ-H, and FMT groups. On day 24, the proportions of CD11b+Ly6G+ and CD11b+Ly6C+ cells were elevated in the Model. CD11b+Ly6G+ cells were reduced in the Cap, YGYZ-H, and the FMT group, whereas no statistical difference was observed in CD11b+Ly6C+ cells (Figure 4B). The results implied that YGYZ depressed the MDSC subpopulation during the pre-metastasis and LM stages.

### 3.3 YGYZ and FMT reduced liver PMN immunosuppressive factors

MDSCs-derived cytokine profiling indicated significant immunosuppressive activity within the hepatic microenvironment during the pre-metastatic phase (Day 12). Compared to Sham group, the Model group exhibited elevated levels of prototypic MDSC-secreted factors, including Arg-1, TGF-β, and IL-10. Cap, YGYZ-H, and FMT groups significantly attenuated these immunosuppressive mediators versus the Model (Figure 4D).

### 3.4 YGYZ and FMT improved the CD4+/CD8+ T cell ratio

The CD4+/CD8+ T cell ratio is a critical indicator of immune function, with low ratios indicating immunosuppression. Flow cytometry revealed that on days 12 and 24, the CD4+/CD8+ T cell ratio decreased in the Model compared with the Sham group. On day 12, YGYZ-H and FMT groups raised the ratio compared with the Model. On day 24, the Cap, YGYZ-H, and FMT groups elevated the ratio compared with the Model, but no statistical difference was seen in the latter two groups (Figure 4C). The findings showed that the primary therapeutic advantage of YGYZ lies in its regulation of IME during the pre-metastatic stage.

### 3.5 YGYZ and FMT ameliorated the GM composition of the LM mouse

**Figure 5:**
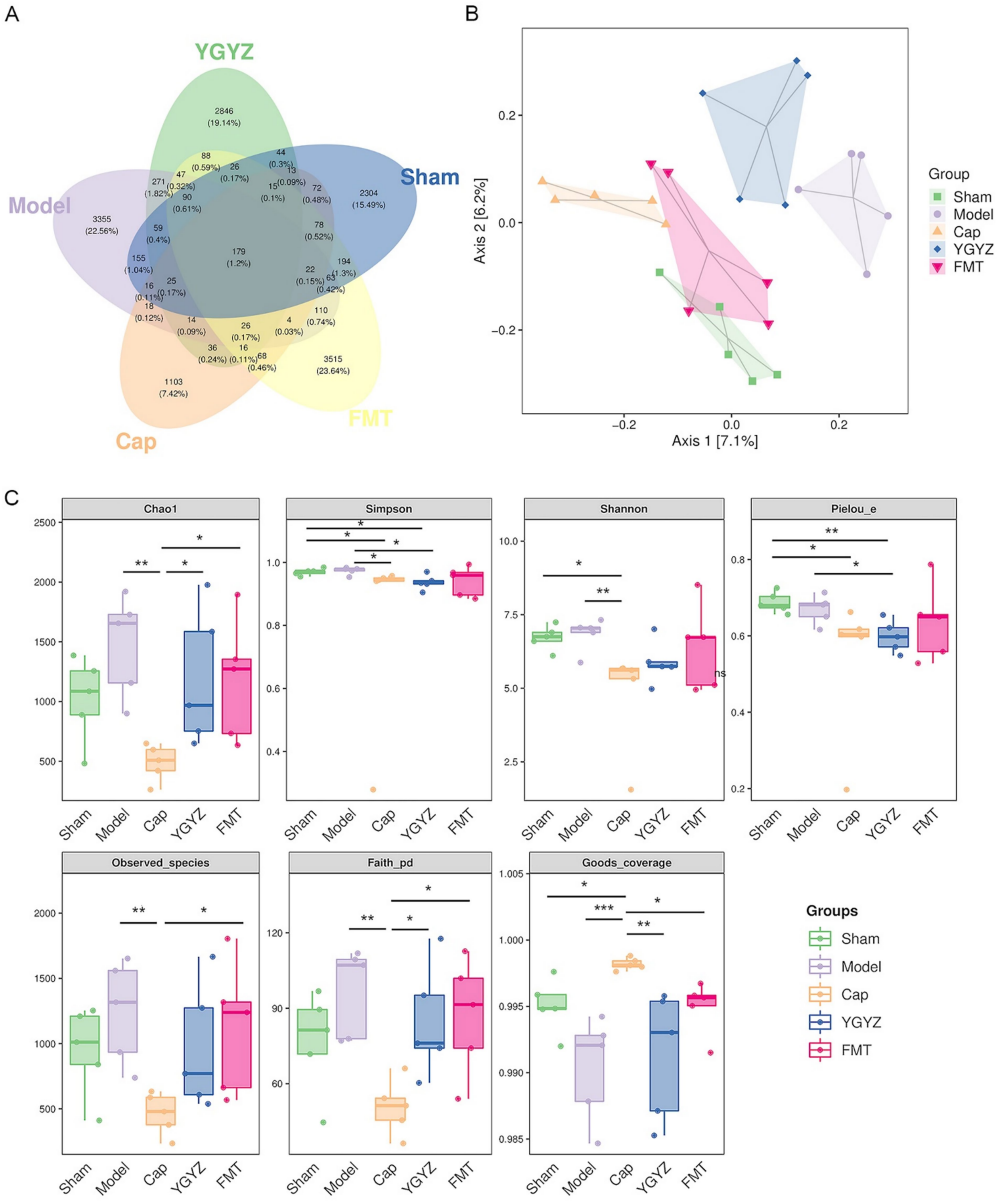
YGYZ and fecal microbiota transplantation impacted the diversity of gut microbiota (GM). **(A)** Venn diagram of amplicon sequence variants/ operational taxonomic unit (*n* = 5). **(B)** Principal coordinates analysis of GM (*n* = 5). **(C)** Alpha diversity indices of GM (*n* = 5). Data are presented as median (IQR) and were statistically analyzed using the Kruskal-Wallis test and multiple comparison Benjamini–Hochberg FDR. *p < 0.05, **p < 0.01, ***p < 0.001.

To investigate differences in GM composition during the pre-metastatic stage, Venn diagram analysis was performed to identify shared and unique operational taxonomic units (OTUs) among five groups. A total of 179 OTUs were shared across all groups, while the Sham, Model, Cap, YGYZ, and FMT groups contained 2,304, 3,355, 1,103, 2,846, and 3,515 unique OTUs, respectively (Figure 5A). Principal coordinate analysis (PCoA) revealed clear separation among the five groups, with PC1 and PC2 explaining 7.1 and 6.2% of the total variance, respectively (Figure 5B). Analysis of the pre-metastatic phase GM demonstrated that the Cap group decreased the index of Chao1, Simpson, Observed species, Shannon, Faith’s PD, and Pielou’s evenness, suggesting that the abundance, diversity, evolutionary diversity, and evenness of the GM declined after the intervention of the chemotherapy (capecitabine). However, there was no difference in α-diversity (Chao1, Observed species, Shannon, Faith’s PD, and Goods coverage) between the YGYZ-H and FMT, with Sham and Model groups, indicating preservation of GM richness, evenness, and diversity (Figure 5C).

**Figure 6:**
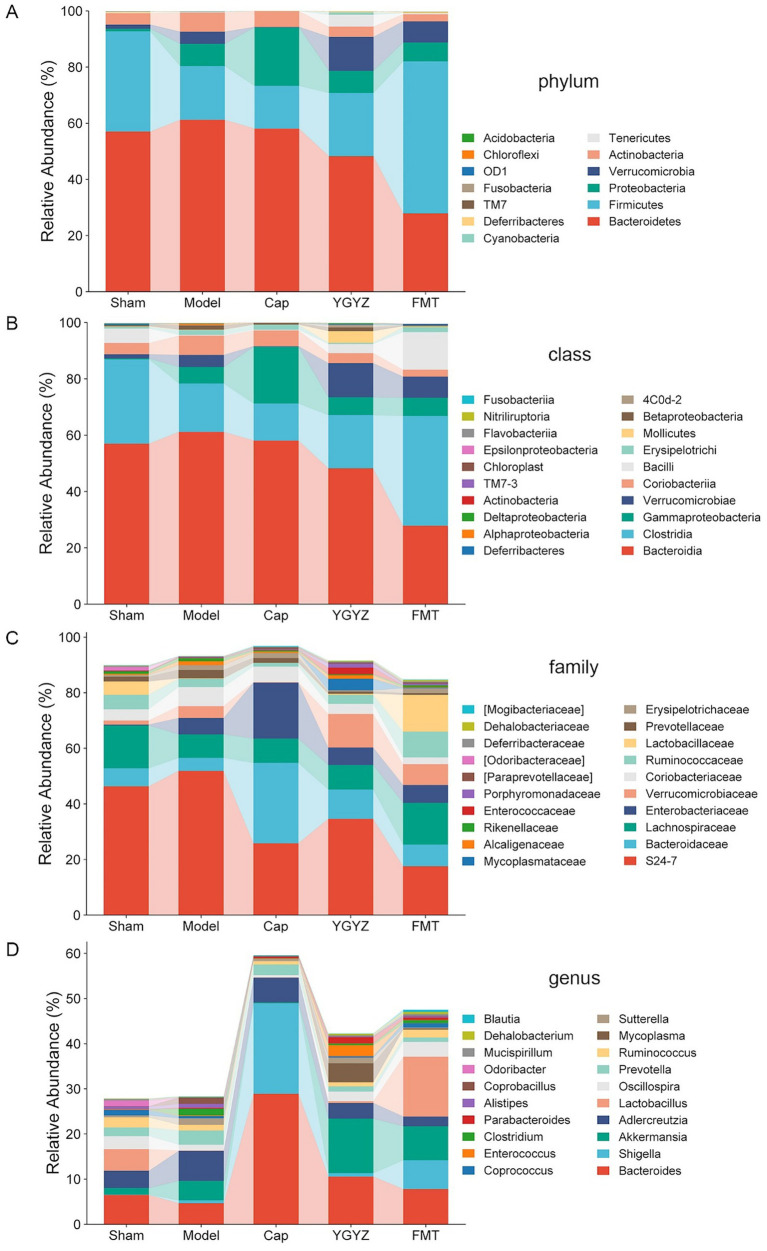
YGYZ impacted the abundance of gut microbiota (GM) at different taxonomic levels. **(A)** Differential relative abundance at the phylum level (*n* = 5). **(B)** Differential relative abundances at the class level (*n* = 5). **(C)** Differential relative abundances at the family level (*n* = 5). **(D)** Differential relative abundances at the genus level (*n* = 5).

Compared to the Model group, both the YGYZ-H and FMT groups showed an increased abundance of Firmicutes and decreased Bacteroidetes, resulting in a higher Firmicutes/Bacteroidetes ratio at the phylum level (Figure 6A). At the class level, the abundance of Bacteroidia decreased, while Clostridia, Verrucomicrobiae, and Bacilli increased (Figure 6B). At the family level, the relative abundance of S24-7 and Coriobacteriaceae was reduced, whereas Bacteroidaceae, Lachnospiraceae, Verrucomicrobiaceae, and Lactobacillaceae were enriched in both treatment groups (Figure 6C). At the genus level, YGYZ-H and FMT exhibited decreased abundance of Adlercreutzia, Prevotella, and increased abundance of Bacteroides, Akkermansia, Lactobacillus, and Oscillospira. In contrast, Cap generated a distinct taxonomic profile divergent from other groups (Figure 6D).

### 3.6 YGYZ reduced the Clostridium abundance of the LM mouse

**Figure 7:**
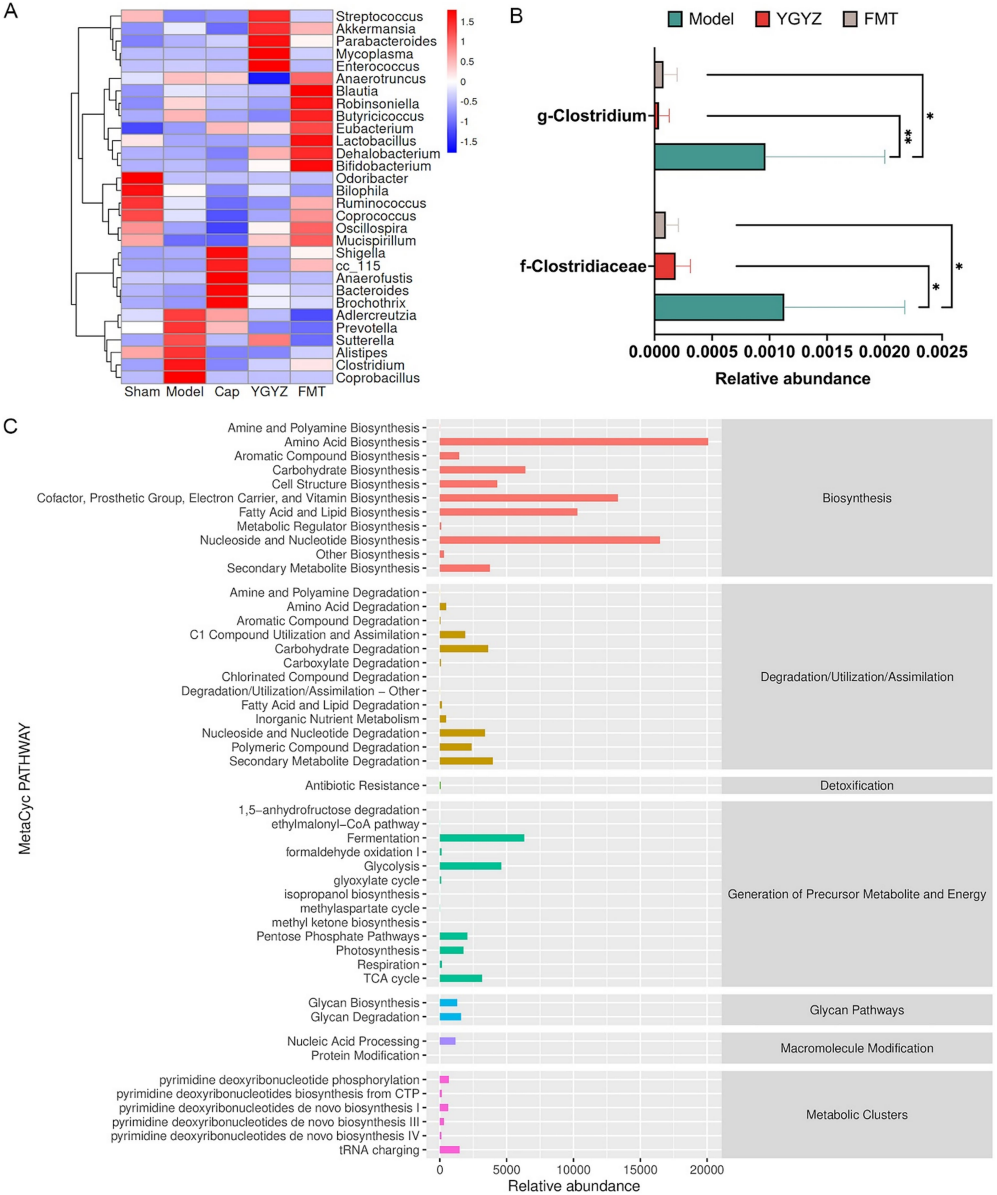
Effects of YGYZ on gut microbiota (GM) composition and function. **(A)** Heatmap showing clustered relative abundance of the top 30 genera at the genus level (red: high abundance, blue: low abundance). **(B)** Modulation of Clostridiaceae and Clostridium abundance by YGYZ and FMT (*n* = 5). **(C)** MetaCyc-based functional profiling of microbial pathways. Data are expressed as mean ± SD. Statistical significance was determined by one-way ANOVA or Kruskal-Wallis test with Benjamini-Hochberg false discovery rate (FDR) correction for multiple comparisons. *p < 0.05, **p < 0.01.

To compare intergroup differences in microbial composition, a clustered heatmap was generated using the top 30 most abundant genera, revealing distinct taxa distribution patterns. Analysis of the heatmap showed that the Model group was enriched in Coprococcus, Clostridium, Alistipes, Sutterella, and Prevotella. Both YGYZ and FMT demonstrated the capacity to reduce these enriched GM genera while increasing the abundance of Akkermansia, Eubacterium, Dehalobacterium, Bifidobacterium, and Mucispirillum (Figure 7A). As Clostridium has been identified as a risk microbiota promoting the progression of CRC (Lin et al., 2025), we quantitatively analyzed their abundance at both family and genus levels. Comparative analysis revealed the Model group maintained higher Clostridium abundance compared to others, which notably reduced in YGYZ-H and FMT groups (Figure 7B). According to the annotation results from the MetaCyc metabolic database, the GM genomes were primarily enriched in biosynthetic pathways, with the highest abundances observed in the biosynthesis of amino acids, nucleotides, prosthetic groups, as well as fatty acids and lipids (Figure 7C). The results suggested an intrinsic connection between GM and BAs.

### 3.7 YGYZ restored the metabolic balance of BAs in the enterohepatic circulation

**Figure 8:**
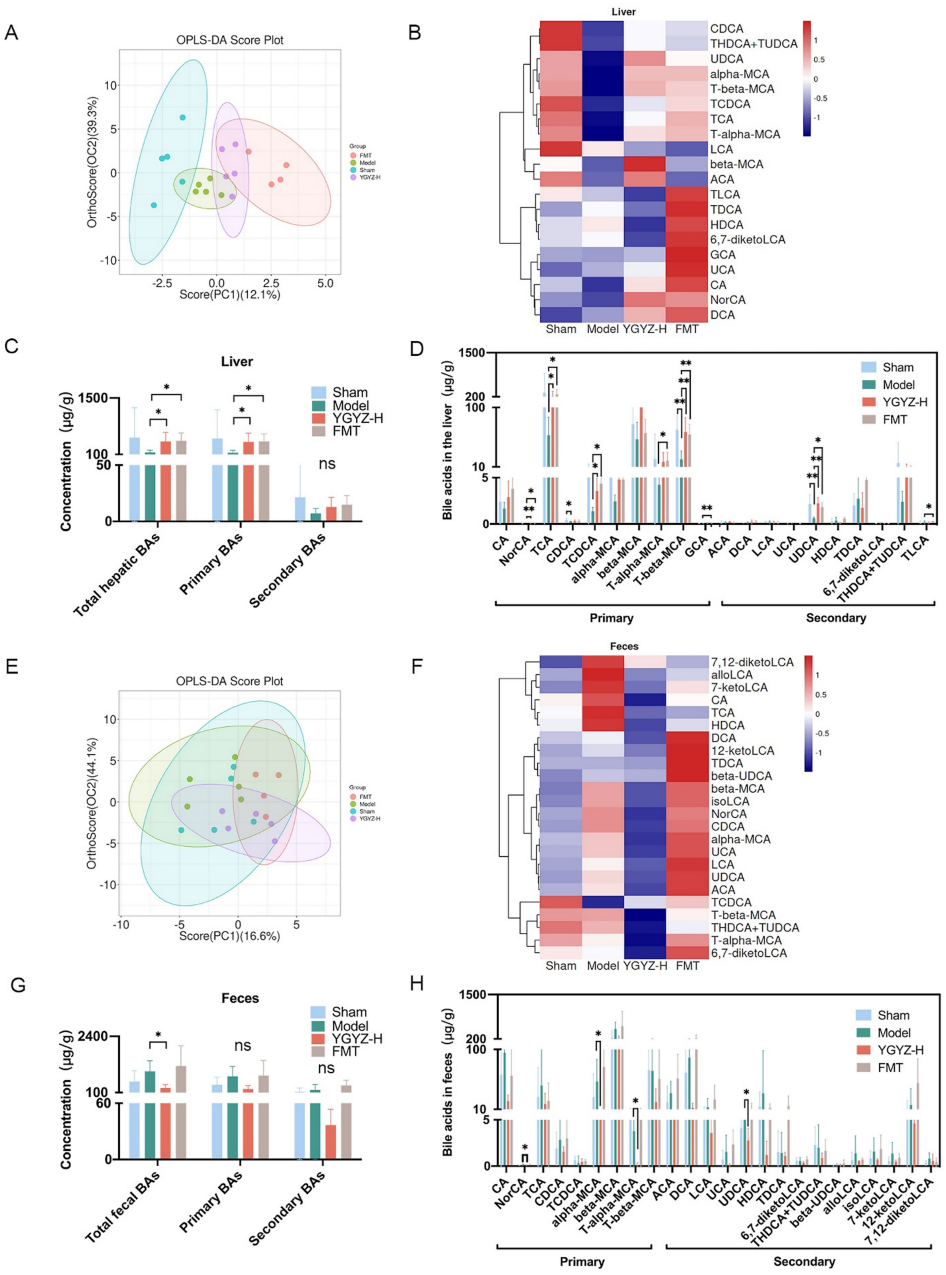
Effects of YGYZ on hepatic and fecal bile acids (BAs) profiles. **(A)** OPLS-DA score plot of hepatic BAs (*n* = 4–5). **(B)** Heatmap of hepatic BAs profiles across the four groups. **(C)** Quantification of total, primary, and secondary BAs in the liver (*n* = 4–5). **(D)** Hepatic BAs metabolic profiles (*n* = 4–5). **(E)** OPLS-DA score plot of fecal BAs (*n* = 4–5). **(F)** Heatmap of fecal BAs profiles across the four groups. **(G)** Quantification of total, primary, and secondary BAs in feces (*n* = 4–5). **(H)** Fecal BAs metabolic profiles (*n* = 4–5). Data are expressed as Mean ± SD using one-way ANOVA or the Kruskal-Wallis test for statistical analysis, Benjamini–Hochberg FDR for multiple comparison corrections. *p < 0.05, **p < 0.01. ns: no significant difference.

Orthogonal Projections to Latent Structures Discriminant Analysis (OPLS-DA) was used to analyze the differences between groups. At the hepatic BAs level, OPLS-DA revealed a separation trend between the Sham, Model, YGYZ-H, and FMT groups, indicating intergroup differences (Figure 8A). Hepatic BAs profiling identified 20 types with >50% detection rates during the pre-metastatic phase. Compared to Sham, the Model exhibited marked BA dysregulation while YGYZ-H and FMT groups restored near-normal profiles (Figure 8B). Compared with the Model, liver total and primary BAs levels were raised in the YGYZ-H and FMT groups, but no difference was seen in secondary BAs among the groups (Figure 8C). Besides, both YGYZ-H and FMT groups enhanced the content of ursodeoxycholic acid (UDCA), Nor cholic acid (NorCA), Taurocholic acid (TCA), Taurochenodeoxycholic Acid (TCDCA), and Tauro beta-Muricholic Acid (T-β-MCA) (Figure 8D).

At the fecal BAs level, OPLS-DA showed no clear separation among the four groups (Figure 8E). Fecal BAs profiling identified 24 metabolites with >50% detection rates during the pre-metastatic phase. The BAs levels of the Model were significantly different from Sham and YGYZ-H groups, which were most similar to the Sham group (Figure 8F). Compared with the Model, fecal total BAs levels were decreased in the YGYZ-H, and no difference was seen in primary and secondary BAs among the groups (Figure 8G). Compared to the Model, YGYZ-H reduced the content of NorCA, alpha-Muricholic acid (α-MCA), Tauro alpha-Muricholic acid (T-α-MCA), and UDCA (Figure 8H). The results suggested that YGYZ restored the metabolic balance of BAs in the enterohepatic circulation.

**Figure 9:**
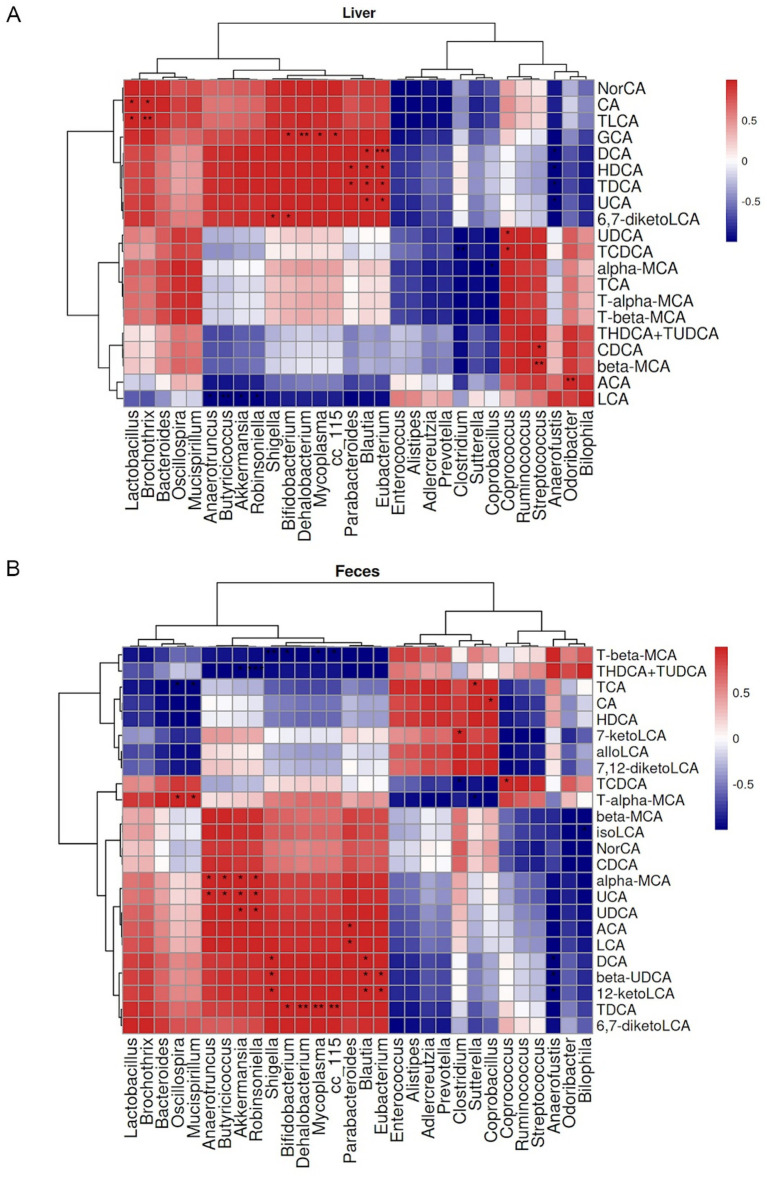
Pearson correlation heatmaps between gut microbiota (GM) at genus level and enterohepatic bile acid (BA) metabolism in mice. **(A)** Heatmap displaying Pearson correlation coefficients between hepatic BAs and GM. **(B)** Heatmap of Pearson correlations between fecal BAs and GM. Red and blue colors indicate positive and negative correlation coefficients, respectively. *p < 0.05, **p < 0.01, ***p < 0.001.

Significant correlations were observed between GM at the genus level and BAs profiles. At the hepatic BAs level, UDCA showed a positive correlation with Coprococcus, and TCDCA was positively correlated with Coprococcus but negatively associated with Clostridium (Figure 9A). In fecal samples, UDCA was positively associated with Akkermansia and Robinsoniella. α-MCA showed positive correlations with Anaerotruncus, Butyricicoccus, Akkermansia, and Robinsoniella. T-α-MCA was positively correlated with Oscillospira and Mucispirillum but negatively associated with Sutterella (Figure 9B).

### 3.8 KLF15 may mediate YGYZ’S regulatory effects on the hepatic BAs-IL-6/STAT3 axis

**Figure 10:**
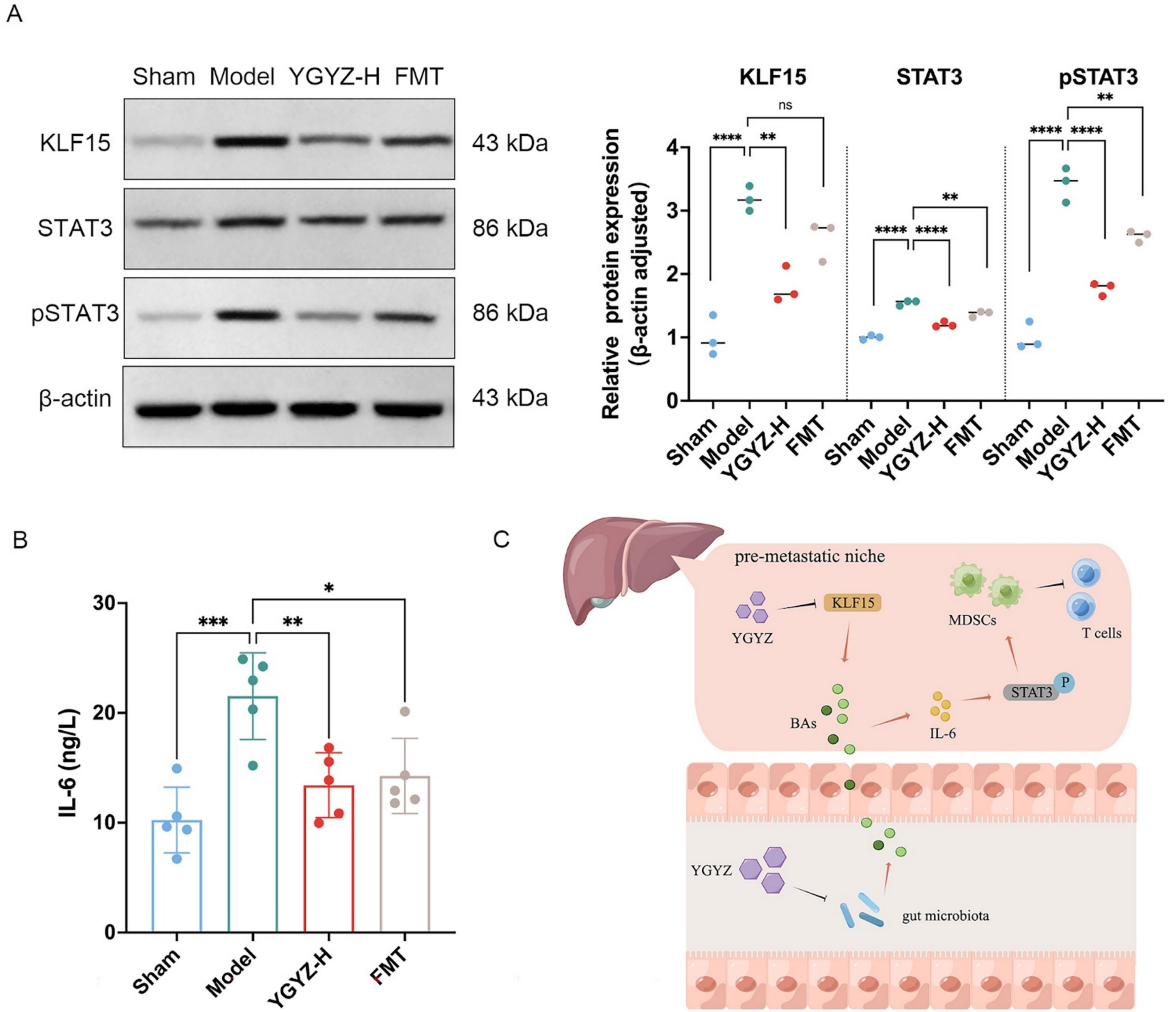
YGYZ downregulated KLF15, STAT3, pSTAT3, and IL-6 protein expression. **(A)** Protein expression levels of hepatic KLF15, total STAT3, and p-STAT3 (*n* = 3). **(B)** Hepatic IL-6 levels measured by ELISA (*n* = 5). **(C)** Schematic diagram illustrating the proposed mechanism by which YGYZ modulated bile acid metabolism and improved the immune microenvironment through gut microbiota and KLF15 regulation. Data are presented as mean ± SD. Statistical analysis was performed using one-way ANOVA and Tukey’s multiple comparisons test. *p < 0.05, **p < 0.01, ***p < 0.001, ****p < 0.0001. ns: no significant difference.

Hepatic KLF15 is a central regulatory node for BAs metabolism (Han et al., 2015), and WB results indicated elevated levels of KLF15 protein expression in the Model against the Sham group. In comparison to the Model, YGYZ-H downregulated KLF15 protein expression, while no difference was seen in the FMT (Figure 8A). The results implied that the KLF15-inhibited function of YGYZ was a synergistic effect of small molecules absorbed into the liver, but not just mediated by GM. Abnormal BAs metabolism directly led to the activation of the IL-6/STAT3 signaling pathway, promoting hepatic inflammation and MDSCs proliferation and activation (Tengesdal et al., 2021). Our study demonstrated that the expression and phosphorylation level of STAT3 protein were most significant in the Model, and both the YGYZ-H and FMT groups depressed hepatic STAT3 and pSTAT3, with pSTAT3 being more pronounced (Figure 10A). The IL-6 protein was expressed significantly in the Model, which was downregulated in both the YGYZ-H and FMT groups (Figure 10B). These results hinted that YGYZ inhibited the IL-6/STAT3 signaling pathway and STAT3 phosphorylation via regulating GM metabolism.

Integrative analysis demonstrated that YGYZ preserved the enterohepatic BAs homeostasis through KLF15 downregulation and GM modulation. This led to suppression of MDSCs proliferation through IL-6/STAT3 pathway, exerting the effect of preventing and controlling LM by strengthening the IME of PMN (Figure 10C).

## 4 Discussion

The liver receives dual blood supply from both the hepatic artery and portal vein with low blood flow velocity, yet the liver sinusoidal endothelial cells possess the most permeable endothelial barrier, creating a particularly favorable microenvironment for cancer cell invasion (Mielgo and Schmid, 2020), explaining why LM represents the most frequent site of distant spread in CRC. In our study, ct26-luc cells were intrasplenically injected and subsequently trafficked via the splenic vein and portal vein into the liver, thereby recapitulating the hematogenous route as the most common pathway for LM. YGYZ effectively prevented LM, promoted early weight gain in tumor-bearing mice, and improved hepatic and renal function, suggesting a favorable safety profile for this decoction. Previous studies demonstrated that Glycyrrhizic acid, a key component of YGYZ, could suppress neutrophil extracellular trap formation, enhancing CD8+ T cell-mediated tumor cytotoxicity and reducing CRC progression (Chen et al., 2025). Bergapten, another active constituent, could enhance p53-mediated apoptotic cascades and suppress AKT phosphorylation, hence decreasing CRC cell viability and triggering cell cycle arrest (Lin et al., 2019). As a crucial component of YGYZ, Icariin can inhibit LM progression by upregulating p53 and Bax proteins while downregulating Bcl-2, thus promoting apoptosis in CRC cells (Zhang et al., 2019).

The IME differs significantly between liver PMN and LM, highlighting the importance of investigating interventions targeting PMNs as a means of preventing LM. MDSCs exert a pivotal role in mediating the formation of liver PMN, being recruited to the liver by tumor-derived secretory factors before cancer cells arrive. Once in the liver, MDSCs actively remodel the IME, facilitating subsequent cancer cell colonization (Conche et al., 2023). Within the PMN, MDSCs impose potent immunosuppressive effects by impairing T-cell responsiveness, promoting M2 macrophage polarization, and facilitating regulatory T cell (Treg) proliferation, all of which foster tumor immune escape (Zeng et al., 2021; Hatziioannou et al., 2017). Notably, PMN-MDSCs are regarded as dominant regulators of the PMN and represent a promising target for novel immunotherapeutic strategies (Wang et al., 2023). Wang et al. illustrated that VEGFA secreted from CRC sites stimulated tumor-associated macrophages to produce chemokine (C-X-C motif) ligand 1 (CXCL1), subsequently mobilizing PMN-MDSCs to the liver via the CXCL1/chemokine (C-X-C motif) receptor 2 (CXCR2) axis, which led to PMN formation and facilitated cancer cell survival in the host organ (Wang et al., 2017). Our study indicated that YGYZ effectively modulated MDSCs accumulation and reduced MDSCs subpopulations during both pre-metastatic and metastatic stages, providing evidence of its preventive and therapeutic efficacy against LM.

The gut and liver are anatomically and functionally interconnected through the portal vein and biliary system, forming the gut-liver axis, which influences maintaining hepatic immune and functional homeostasis (Tian et al., 2023). The interaction between BAs and the GM has been identified as a key factor in promoting LM, making it a potential therapeutic target for LM treatment (Ma et al., 2018). GM is predominantly composed of five major phyla: Firmicutes, Bacteroidetes, Proteobacteria, Verrucomicrobia, and Actinobacteria, with the former two collectively accounting for 90–95% of the total abundance (Morris et al., 2023). At the phylum level, multiple clinical studies have detected that CRC patients exhibited an increased abundance of Bacteroidetes but a decreased abundance of Firmicutes (Villéger et al., 2018), and the Firmicutes/Bacteroidetes (F/B) ratio has been proposed as a potential biomarker for gut dysbiosis in CRC (Di Pierro, 2021). In line with these observations, our model group exhibited a significantly reduced F/B ratio, while YGYZ and FMT reversed this dysbiotic signature by increasing Firmicutes and decreasing Bacteroidetes. Epidemiological studies have consistently linked Clostridioides difficile (*C. difficile*) to elevated risks of increasing incidence and mortality of CRC (Bassotti et al., 2023), and evidence suggests that host-produced primary BAs serve as the dominant germination signals (Anderson and Sears, 2023). Using three murine models (subcutaneous, intrasplenic, and tail vein injection of tumor cells), they observed Clostridium colonization resulted in a reduction of primary BAs and induced a rapid reduction of hepatic NKT cells, which suppressed anti-tumor immunity and promoted cancer colonization and survival (Ma et al., 2018). In our study, we observed a marked enrichment of Clostridium in the Model group, while YGYZ significantly reduced its abundance in LM mice. These findings were consistent in the FMT group, confirming successful microbial modulation. Previous studies have demonstrated that FMT could modulate anti-tumor immunity in MSS-type CRC tumor-bearing mice, showing promising therapeutic potential (Yue et al., 2020). Yu et al. revealed that FMT restored GM balance in CRC mice, promoting the infiltration of CD8^+^ T cells, CD4^+^ T cells, and CD49b^+^ NK cells while suppressing Foxp3^+^ Treg cells, thereby effectively inhibiting CRC progression (Yu et al., 2023). Routy et al. reported that in germ-free mice receiving FMT from immunotherapy-refractory patients, oral administration of Akkermansia reinstated the anti-tumor effects of immune checkpoint inhibitors (ICI). Mechanistically, Akkermansia induced IL-12 production, which promoted the infiltration of CCR9^+^CXCR3^+^CD4^+^ T lymphocytes into the tumor microenvironment, thereby rescuing ICI responsiveness (Routy et al., 2018). In this study, YGYZ and FMT treatment reduced pathogenic bacteria (e.g., Clostridium) while increasing beneficial microbes such as Akkermansia. Concurrently, they suppressed hepatic PMN-MDSCs and immunosuppressive factors, restored the CD4^+^/CD8^+^ T cell ratio, and reprogrammed the liver PMN.

Dysregulated BAs metabolism has been identified as a risk factor for CRC development and metastasis. Approximately 95% of BAs are absorbed in the distal ileum and re-enter the enterohepatic circulation, while the remaining 5% serve as substrates for GM metabolism, where they are converted into secondary BAs by bacterial bile salt hydrolase activity. These secondary BAs participate in various physiological and pathological processes (Zeng et al., 2019). Farhana et al. discovered that unconjugated secondary BAs, represented by deoxycholic acid (DCA) and lithocholic acid, induced colorectal carcinogenesis by stimulating genomic instability and epithelial-mesenchymal transition (EMT) in colonic epithelial cells, ultimately driving their transformation into CRC stem-like cells (Farhana et al., 2016). Moreover, BAs have been indicated to facilitate LM. Deng et al. demonstrated that antibiotic treatment specifically suppressed LM, without affecting lung metastasis or subcutaneous flank tumors. Antibiotics reduced the abundance of Clostridium cluster XIVa and decreased DCA levels, highlighting the critical role of GM and BA metabolism in promoting LM (Deng et al., 2022). In our study, YGYZ significantly decreased fecal BA levels while increasing hepatic BA content in the Model, effectively restoring the enterohepatic circulation to a state similar to the Sham group. FMT demonstrated a comparable effect in elevating hepatic BA levels. These findings collectively suggest that YGYZ may exert its beneficial effects through targeted modulation of GM composition, subsequently reestablishing BAs homeostasis. Specifically, YGYZ markedly elevated hepatic UDCA and concurrently decreased fecal levels. Recent studies revealed that UDCA imposed anti-tumor effects through multiple pathways via targeting colon cancer cells by downregulating c-Myc protein expression, thereby suppressing proliferation and inducing apoptosis (Peiró-Jordán et al., 2012). At low concentrations, UDCA suppressed oncogenic signaling transduction of the inflammation-driven CRC mouse model, demonstrating chemopreventive potential (Khare et al., 2008). In addition, UDCA might exert immunomodulatory activity through blocking Treg cell differentiation and activation in tumor-bearing hosts by TGF-β degradation, enhancing anti-tumor immunity (Shen et al., 2022). However, clinical evidence paradoxically associated high-dose UDCA supplementation with rising CRC risk (Lindström et al., 2012), implying the ongoing controversy surrounding its dose-dependent effects. Additionally, our study demonstrated that YGYZ modulated the enterohepatic circulation of MCAs and facilitated the restoration of BAs homeostasis. MCAs constitute the predominant BAs in mice, with T-α-MCA and T-β-MCA representing taurine-conjugated primary BAs. In LM models, antibiotic treatment significantly suppressed metastatic progression while concurrently elevating hepatic levels of the primary BAs T-β-MCA and β-MCA (Sayin et al., 2013).

KLF15 serves as a central regulator of endogenous metabolism and maintains the BAs pool homeostasis by orchestrating BAs synthesis, transport, and metabolic processes in the liver (Wang et al., 2020). Current findings establish KLF15 as a master regulator coordinating the expression of BAs biosynthesis enzymes, BAs pool dynamics, and intestinal fat absorption (Chen et al., 2023). Research has revealed that ileal fibroblast growth factor 15 (FGF15) acts as a suppressor of BA synthesis. In the intestine, BAs activate farnesoid X receptor (FXR) to stimulate FGF15 production, establishing a negative feedback loop that inhibits hepatic BAs synthesis (Han et al., 2015). Concurrently, the KLF15/FGF15 pathway regulates the transcription of key genes involved in BA biosynthesis (such as CYP7A1 and CYP8B1) in hepatocytes. This pathway modulates BAs production by repressing rate-limiting enzymes in the biosynthesis pathway, including cholesterol 7α-hydroxylase (Wang et al., 2020). However, systematic investigations into the quantitative relationship between KLF15 expression patterns and individual BAs species are still lacking. In this study, YGYZ lowered KLF15 expression in the liver, whereas no significant change was observed in the FMT group compared to the Model. Therefore, it was speculated that YGYZ-derived small molecules absorbed into hepatic circulation, modulated BAs metabolism and coordinately regulated the PMN via downregulating KLF15. Aberrant BAs metabolism directly modulates hepatic immune signaling. In both bile duct-ligated mice and patients with obstructive cholestasis, marked liver inflammation was observed, with elevated levels of pro-inflammatory cytokines (IL-6, IL-1β, and tumor necrosis factor-α). Enhanced STAT3 phosphorylation has been identified as a central mechanism by which BAs mediate hepatic inflammation (Roh et al., 2018; Chai et al., 2012). Our results concluded that YGYZ downregulated hepatic KLF15 expression during the pre-metastatic period and remodeled GM synergistically. These combined effects helped restore the enterohepatic BAs cycle and suppress activation of the IL-6/STAT3 pathway. As a result, YGYZ inhibited MDSCs’ proliferation and activation, thereby attenuating PMN formation and effectively preventing LM.

## 5 Conclusion

In summary, YGYZ effectively prevented LM and modulated hepatic PMN with a favorable safety profile. Mechanistically, YGYZ might reshaped GM composition and suppressed KLF15 to repair enterohepatic BAs dysregulation, thereby repressing IL-6/STAT3-driven MDSCs expansion and activation. This multi-modal action reshaped the immunosuppressive PMN into a metastasis-restrictive microenvironment in the liver. These findings not only elucidate the mechanistic basis underlying the TCM principle of “liver–spleen coordination” but also propose GM and BAs profiles as promising predictive biomarkers for LM risk. From a clinical perspective, YGYZ represents a safe and promising complementary strategy that could be integrated into current therapeutic regimens to prevent LM and modulate the PMN.

However, this study has several limitations that warrant further investigation. The quantitative relationship between KLF15 expression levels and GM composition/ BAs metabolic profiles remains unclear, necessitating deeper exploration of its regulatory network. Although FMT experiments demonstrated anti-metastatic effects of microbiota transplantation, the impact of transplantation frequency on recipient microbiota colonization stability requires systematic evaluation. Additionally, the key active components in the herbal formulation targeting KLF15 and IL-6/STAT3 pathways, along with their synergistic mechanisms, demand further elucidation. Future studies should focus on elucidating KLF15’s interaction with the GM-BAs axis through correlation analysis and KLF15 knockdown experiments, optimizing FMT protocols by assessing frequency-dependent effects on bacterial colonization stability, isolating and characterizing bioactive components to establish component-target-pathway networks, and conducting pharmacokinetics and Pharmacodynamics studies followed by clinical trials to facilitate translational application of these findings.

## Data Availability

The original contributions presented in the study are included in the article/Supplementary material, further inquiries can be directed to the corresponding author.

## Footnotes

^1^ https://metacyc.org/

## Glossary

CRC: Colorectal cancer
LM: Liver metastasis
PMN: Pre-metastatic niche
MDSCs: Myeloid-derived suppressor cells
GM: Gut microbiota
BAs: Bile acids
TCM: Traditional Chinese medicine
YGYZ: Yanggan Yizhong
UPLC-HR-MS/MS: Ultra-performance liquid chromatography-high resolution tandem mass spectrometry
FMT: Fecal microbiota transplantation
HE: Hematoxylin and eosin
ALT: Alanine aminotransferase
AST: Aspartate aminotransferase
BUN: Blood urea nitrogen
CRE: Creatinine
RT: Room temperature
ELISA: Enzyme-linked immunosorbent assay
Arg-1: Arginase-1
TGF-β: Transforming growth factor-β
IL-10: Interleukin-10
IL-6: Interleukin-6
WB: Western blot
KLF15: Kruppel-like factor 15
IME: Immune microenvironment
OUTs: Operational taxonomic units
OPLS-DA: Orthogonal projections to latent structures discriminant analysis
UDCA: Ursodeoxycholic acid
NorCA: Nor cholic acid
TCA: Taurocholic acid
TCDCA: Taurochenodeoxycholic acid
T-β-MCA: Tauro beta-muricholic acid
α-MCA: Alpha-muricholic acid
T-α-MCA: Tauro alpha-muricholic acid
Treg: Regulatory T cell
CXCL1: Chemokine (C-X-C motif) ligand 1
CXCR2: Chemokine (C-X-C motif) receptor 2
F/B: Firmicutes/bacteroidetes
DCA: Deoxycholic acid
EMT: Epithelial-mesenchymal transition
ICI: Immune checkpoint inhibitors
FGF15: Fibroblast growth factor 15
FXR: Farnesoid X receptor

## References

[ref1] Anderson S. M. Sears C. L. (2023). The role of the gut microbiome in cancer: a review, with special focus on colorectal neoplasia and *Clostridioides difficile*. Clin. Infect. Dis. 77, S471–S478. doi: 10.1093/cid/ciad640, PMID: 38051969 PMC10697667

[ref2] Aykut B. Lidsky M. E. (2023). Colorectal Cancer liver metastases: multimodal therapy. Surg. Oncol. Clin. N. Am. 32, 119–141. doi: 10.1016/j.soc.2022.07.009, PMID: 36410912

[ref3] Bassotti G. Stracci F. Marconi P. Fettucciari K. (2023). Clostridioides difficile and colorectal cancer: a dangerous liaison. Eur. J. Gastroenterol. Hepatol. 35, 985–988. doi: 10.1097/meg.0000000000002615, PMID: 37505976

[ref4] Bray F. Laversanne M. Sung H. Ferlay J. Siegel R. L. Soerjomataram I.. (2024). Global cancer statistics 2022: GLOBOCAN estimates of incidence and mortality worldwide for 36 cancers in 185 countries. CA Cancer J. Clin. 74, 229–263. doi: 10.3322/caac.21834, PMID: 38572751

[ref5] Chai J. He Y. Cai S. Y. Jiang Z. Wang H. Li Q.. (2012). Elevated hepatic multidrug resistance-associated protein 3/ATP-binding cassette subfamily C 3 expression in human obstructive cholestasis is mediated through tumor necrosis factor alpha and c-Jun NH2-terminal kinase/stress-activated protein kinase-signaling pathway. Hepatology 55, 1485–1494. doi: 10.1002/hep.24801, PMID: 22105759 PMC3297707

[ref6] Chen H. Li L. L. Du Y. (2023). Krüppel-like factor 15 in liver diseases: insights into metabolic reprogramming. Front. Pharmacol. 14:1115226. doi: 10.3389/fphar.2023.1115226, PMID: 36937859 PMC10017497

[ref7] Chen Y. L. Xu B. Pan Z. F. Cai Y. P. Yang C. Y. Cao S. L.. (2025). Glycyrrhizic acid reduces neutrophil extracellular trap formation to ameliorate colitis-associated colorectal cancer by inhibiting peptidylarginine deiminase 4. J. Ethnopharmacol. 341:119337. doi: 10.1016/j.jep.2025.119337, PMID: 39788166

[ref8] Conche C. Finkelmeier F. Pešić M. Nicolas A. M. Böttger T. W. Kennel K. B.. (2023). Combining ferroptosis induction with MDSC blockade renders primary tumours and metastases in liver sensitive to immune checkpoint blockade. Gut 72, 1774–1782. doi: 10.1136/gutjnl-2022-327909, PMID: 36707233 PMC10423492

[ref9] Deng J. Yuan W. Tan Q. Wei X. Ma J. (2022). Non-absorbable antibiotic treatment inhibits colorectal cancer liver metastasis by modulating deoxycholic acid metabolism by intestinal microbes. J. Cancer 13, 764–774. doi: 10.7150/jca.63490, PMID: 35154445 PMC8824892

[ref10] Di Pierro F. (2021). Gut microbiota parameters potentially useful in clinical perspective. Microorganisms 9:2402. doi: 10.3390/microorganisms9112402, PMID: 34835527 PMC8623243

[ref11] Eng C. Jácome A. A. Agarwal R. Hayat M. H. Byndloss M. X. Holowatyj A. N.. (2022). A comprehensive framework for early-onset colorectal cancer research. Lancet Oncol. 23, e116–e128. doi: 10.1016/s1470-2045(21)00588-x, PMID: 35090673

[ref12] Farhana L. Nangia-Makker P. Arbit E. Shango K. Sarkar S. Mahmud H.. (2016). Bile acid: a potential inducer of colon cancer stem cells. Stem Cell Res Ther 7:181. doi: 10.1186/s13287-016-0439-4, PMID: 27908290 PMC5134122

[ref13] Han S. Zhang R. Jain R. Shi H. Zhang L. Zhou G.. (2015). Circadian control of bile acid synthesis by a KLF15-Fgf15 axis. Nat. Commun. 6:7231. doi: 10.1038/ncomms8231, PMID: 26040986 PMC4457302

[ref14] Hatziioannou A. Alissafi T. Verginis P. (2017). Myeloid-derived suppressor cells and T regulatory cells in tumors: unraveling the dark side of the force. J. Leukoc. Biol. 102, 407–421. doi: 10.1189/jlb.5VMR1116-493R, PMID: 28360184

[ref15] Kaplan R. N. Riba R. D. Zacharoulis S. Bramley A. H. Vincent L. Costa C.. (2005). VEGFR1-positive haematopoietic bone marrow progenitors initiate the pre-metastatic niche. Nature 438, 820–827. doi: 10.1038/nature04186, PMID: 16341007 PMC2945882

[ref16] Kaviyarasan V. Das A. Deka D. Saha B. Banerjee A. Sharma N. R.. (2024). Advancements in immunotherapy for colorectal cancer treatment: a comprehensive review of strategies, challenges, and future prospective. Int. J. Color. Dis. 40:1. doi: 10.1007/s00384-024-04790-w, PMID: 39731596 PMC11682016

[ref17] Khare S. Mustafi R. Cerda S. Yuan W. Jagadeeswaran S. Dougherty U.. (2008). Ursodeoxycholic acid suppresses Cox-2 expression in colon cancer: roles of Ras, p38, and CCAAT/enhancer-binding protein. Nutr. Cancer 60, 389–400. doi: 10.1080/01635580701883003, PMID: 18444174

[ref18] Lin H. Chen Y. Zhou M. Wang H. Chen L. Zheng L.. (2025). Comprehensive analysis of faecal metagenomic and serum metabolism revealed the role of gut microbes and related metabolites in detecting colorectal lateral spreading tumours. Virulence 16:2489154. doi: 10.1080/21505594.2025.2489154, PMID: 40223231 PMC12005448

[ref19] Lin C. P. Lin C. S. Lin H. H. Li K. T. Kao S. H. Tsao S. M. (2019). Bergapten induces G1 arrest and pro-apoptotic cascade in colorectal cancer cells associating with p53/p21/PTEN axis. Environ. Toxicol. 34, 303–311. doi: 10.1002/tox.22685, PMID: 30576070

[ref20] Lindström L. Boberg K. M. Wikman O. Friis-Liby I. Hultcrantz R. Prytz H.. (2012). High dose ursodeoxycholic acid in primary sclerosing cholangitis does not prevent colorectal neoplasia. Aliment. Pharmacol. Ther. 35, 451–457. doi: 10.1111/j.1365-2036.2011.04966.x 22221173

[ref21] Ma C. Han M. Heinrich B. Fu Q. Zhang Q. Sandhu M.. (2018). Gut microbiome-mediated bile acid metabolism regulates liver cancer via NKT cells. Science 360:eaan5931. doi: 10.1126/science.aan5931, PMID: 29798856 PMC6407885

[ref22] Mielgo A. Schmid M. C. (2020). Liver tropism in Cancer: the hepatic metastatic niche. Cold Spring Harb. Perspect. Med. 10:3. doi: 10.1101/cshperspect.a037259, PMID: 31548227 PMC7050581

[ref23] Morris M. T. Jain A. Sun B. Kurbatov V. Muca E. Zeng Z.. (2023). Multi-omic analysis reveals metabolic pathways that characterize right-sided colon cancer liver metastasis. Cancer Lett. 574:216384. doi: 10.1016/j.canlet.2023.216384, PMID: 37716465 PMC10620771

[ref24] Nair A. B. Jacob S. (2016). A simple practice guide for dose conversion between animals and human. J. Basic Clin. Pharm. 7, 27–31. doi: 10.4103/0976-0105.177703, PMID: 27057123 PMC4804402

[ref25] Peiró-Jordán R. Krishna-Subramanian S. Hanski M. L. Lüscher-Firzlaff J. Zeitz M. Hanski C. (2012). The chemopreventive agent ursodeoxycholic acid inhibits proliferation of colon carcinoma cells by suppressing c-Myc expression. Eur. J. Cancer Prev. 21, 413–422. doi: 10.1097/CEJ.0b013e32834ef16f, PMID: 22395148

[ref26] Roh Y. S. Cho A. Cha Y. S. Oh S. H. Lim C. W. Kim B. (2018). Lactobacillus aggravate bile duct ligation-induced liver inflammation and fibrosis in mice. Toxicol. Res. 34, 241–247. doi: 10.5487/tr.2018.34.3.241, PMID: 30057698 PMC6057294

[ref27] Routy B. Le Chatelier E. Derosa L. Duong C. P. M. Alou M. T. Daillère R.. (2018). Gut microbiome influences efficacy of PD-1-based immunotherapy against epithelial tumors. Science 359, 91–97. doi: 10.1126/science.aan3706 29097494

[ref28] Sayin S. I. Wahlström A. Felin J. Jäntti S. Marschall H. U. Bamberg K.. (2013). Gut microbiota regulates bile acid metabolism by reducing the levels of tauro-beta-muricholic acid, a naturally occurring FXR antagonist. Cell Metab. 17, 225–235. doi: 10.1016/j.cmet.2013.01.003 23395169

[ref29] Shen Y. Lu C. Song Z. Qiao C. Wang J. Chen J.. (2022). Ursodeoxycholic acid reduces antitumor immunosuppression by inducing CHIP-mediated TGF-β degradation. Nat. Commun. 13:3419. doi: 10.1038/s41467-022-31141-6, PMID: 35701426 PMC9198048

[ref30] Stewart C. L. Warner S. Ito K. Raoof M. Wu G. X. Kessler J.. (2018). Cytoreduction for colorectal metastases: liver, lung, peritoneum, lymph nodes, bone, brain. When does it palliate, prolong survival, and potentially cure? Curr. Probl. Surg. 55, 330–379. doi: 10.1067/j.cpsurg.2018.08.004, PMID: 30526930 PMC6422355

[ref31] Tengesdal I. W. Dinarello A. Powers N. E. Burchill M. A. Joosten L. A. B. Marchetti C.. (2021). Tumor NLRP3-derived IL-1β drives the IL-6/STAT3 Axis resulting in sustained MDSC-mediated immunosuppression. Front. Immunol. 12:661323. doi: 10.3389/fimmu.2021.661323, PMID: 34531850 PMC8438323

[ref32] Tian P. Yang W. Guo X. Wang T. Tan S. Sun R.. (2023). Early life gut microbiota sustains liver-resident natural killer cells maturation via the butyrate-IL-18 axis. Nat. Commun. 14:1710. doi: 10.1038/s41467-023-37419-7, PMID: 36973277 PMC10043027

[ref33] Villéger R. Lopès A. Veziant J. Gagnière J. Barnich N. Billard E.. (2018). Microbial markers in colorectal cancer detection and/or prognosis. World J. Gastroenterol. 24, 2327–2347. doi: 10.3748/wjg.v24.i22.2327, PMID: 29904241 PMC6000297

[ref34] Wang D. Sun H. Wei J. Cen B. DuBois R. N. (2017). CXCL1 is critical for Premetastatic niche formation and metastasis in colorectal Cancer. Cancer Res. 77, 3655–3665. doi: 10.1158/0008-5472.Can-16-3199, PMID: 28455419 PMC5877403

[ref35] Wang G. Wu B. Cui Y. Zhang B. Jiang C. Wang H. (2020). Teneligliptin promotes bile acid synthesis and attenuates lipid accumulation in obese mice by targeting the KLF15-Fgf15 pathway. Chem. Res. Toxicol. 33, 2164–2171. doi: 10.1021/acs.chemrestox.0c00192 32639145

[ref36] Wang C. Zheng X. Zhang J. Jiang X. Wang J. Li Y.. (2023). CD300ld on neutrophils is required for tumour-driven immune suppression. Nature 621, 830–839. doi: 10.1038/s41586-023-06511-9, PMID: 37674079

[ref37] Wu Z. Huang S. Li T. Li N. Han D. Zhang B.. (2021). Gut microbiota from green tea polyphenol-dosed mice improves intestinal epithelial homeostasis and ameliorates experimental colitis. Microbiome 9:184. doi: 10.1186/s40168-021-01115-9, PMID: 34493333 PMC8424887

[ref38] Xu Y. Zhang L. Wang Q. Zheng M. (2020). Comparison of different colorectal cancer with liver metastases models using six colorectal cancer cell lines. Pathol. Oncol. Res. 26, 2177–2183. doi: 10.1007/s12253-020-00805-3, PMID: 32172478

[ref39] Yu H. Li X. X. Han X. Chen B. X. Zhang X. H. Gao S.. (2023). Fecal microbiota transplantation inhibits colorectal cancer progression: reversing intestinal microbial dysbiosis to enhance anti-cancer immune responses. Front. Microbiol. 14:1126808. doi: 10.3389/fmicb.2023.1126808, PMID: 37143538 PMC10151806

[ref40] Yue Y. C. Yang B. Y. Lu J. Zhang S. W. Liu L. Nassar K.. (2020). Metabolite secretions of *Lactobacillus plantarum* YYC-3 may inhibit colon cancer cell metastasis by suppressing the VEGF-MMP2/9 signaling pathway. Microb. Cell Factories 19:213. doi: 10.1186/s12934-020-01466-2, PMID: 33228670 PMC7684877

[ref41] Zeng H. Umar S. Rust B. Lazarova D. Bordonaro M. (2019). Secondary bile acids and short chain fatty acids in the Colon: a focus on colonic microbiome, cell proliferation, inflammation, and Cancer. Int. J. Mol. Sci. 20:1214. doi: 10.3390/ijms20051214, PMID: 30862015 PMC6429521

[ref42] Zeng D. Wang M. Wu J. Lin S. Ye Z. Zhou R.. (2021). Immunosuppressive microenvironment revealed by immune cell landscape in pre-metastatic liver of colorectal Cancer. Front. Oncol. 11:620688. doi: 10.3389/fonc.2021.620688, PMID: 33833986 PMC8021849

[ref43] Zhang L. Qiao D. Mao H. Li Y. Dong X. Kong G.. (2019). Study on the inhibitory effect and mechanism of icariin on liver metastasis of colon cancer in mice. Lishizhen Med. Mater. Med. Res. 30, 1829–1832. doi: 10.3969/j.issn.1008-0805.2019.08.013

[ref44] Zhao L. Zhu X. Ni Y. You J. Li A. (2020). Xiaoyaosan, a traditional Chinese medicine, inhibits the chronic restraint stress-induced liver metastasis of colon cancer *in vivo*. Pharm. Biol. 58, 1085–1091. doi: 10.1080/13880209.2020.1839513, PMID: 33152259 PMC7646552

[ref45] Zhou H. Liu Z. Wang Y. Wen X. Amador E. H. Yuan L.. (2022). Colorectal liver metastasis: molecular mechanism and interventional therapy. Signal Transduct. Target. Ther. 7:70. doi: 10.1038/s41392-022-00922-2, PMID: 35246503 PMC8897452

[ref46] Zong X. Zhang H. Zhu L. Deehan E. C. Fu J. Wang Y.. (2023). Auricularia auricula polysaccharides attenuate obesity in mice through gut commensal *Papillibacter cinnamivorans*. J. Adv. Res. 52, 203–218. doi: 10.1016/j.jare.2023.08.003, PMID: 37549868 PMC10555930

